# Health-related quality of life after spontaneous subarachnoid hemorrhage – a prospective cohort study

**DOI:** 10.1007/s11136-025-03955-6

**Published:** 2025-03-27

**Authors:** Verena Rass, Anna Berek, Klaus Altmann, Elisabeth Goettfried, Philipp Kindl, Raimund Helbok, Alois Schiefecker, Bettina Pfausler, Laura Zamarian, Ronny Beer

**Affiliations:** 1 Department of Neurology, Medical University of Innsbruck, Anichstrasse 35, 6020 Innsbruck, Austria; 2 Department of Neurology, General Hospital Barmherzige Schwestern, Ried Im Innkreis, Austria; 3 Department of Neurology, Kepler University Hospital, Johannes Kepler University Linz, Linz, Austria; 4 Clinical Research Institute of Neuroscience, Johannes Kepler University Linz, Kepler University Hospital, Linz, Austria

**Keywords:** Subarachnoid haemorrhage, Quality of life, Outcome, Mental health

## Abstract

**Purpose:**

Reduced health-related quality of life (HR-QoL) is common after spontaneous subarachnoid hemorrhage (SAH). Here, we aimed to describe the prevalence of HR-QoL impairment one year after SAH and to identify associated factors.

**Methods:**

In this prospective cohort study, HR-QoL was assessed in 183 patients one year after SAH. We used the Short-Form-36 (SF-36) questionnaire, which consists of eight health domains that can be subdivided into mental and physical health components. Participants responded to scales on subjective attention deficit, mental health symptoms, and fatigue. Functional outcome was assessed with the modified Rankin Scale (mRS). Multivariable regression analysis was used to identify factors associated with reduced HR-QoL (MCS or PCS < 40).

**Results:**

Patients were 53 years of age (IQR, 46–61) and presented with a median Hunt&Hess score of 2 (2–3). HR-QoL was reduced in 66/183 patients (36%) with the highest abnormality in physical and emotional role. A lower Hunt&Hess score (p = 0.036), female sex (p = 0.017), self-reported depression (p = 0.001), fatigue (p < 0.001), and reduction of drive (p = 0.019) were associated with overall reduced HR-QoL and explained 68.9% of the observed variance. 26% (n = 48) scored below the normal range on the MCS, and independent associations emerged for self-reported anxiety and depression, fatigue, and reduction of drive. A reduction in the PCS was reported by 35 (19%) patients and independent associations were found for worse three-month functional outcome and fatigue.

**Conclusion:**

One in three patients reported a reduction in HR-QoL one year after SAH. Mental health problems and fatigue had a significant impact on HR-QoL.

**Supplementary Information:**

The online version contains supplementary material available at 10.1007/s11136-025-03955-6.

## Introduction

Spontaneous subarachnoid hemorrhage (SAH) is characterised by bleeding in the subarachnoid space and is associated with severe headache. An aneurysm is the underlying source of bleeding in most patients, but in about 15% of patients the source of bleeding cannot be identified on angiography [1]. SAH accounts for only 2–5% of strokes [2]. The incidence of SAH is 1.3 times higher in women than in men and increases with age [3]. Despite advances in critical care management and improved prognosis, SAH remains associated with high morbidity and mortality [4]. The 90-day mortality rate for patients hospitalised with SAH is approximately 30% [4]. Survivors often suffer from functional and cognitive sequelae, fatigue, mental health and psychosocial problems, including depression and anxiety, affecting up to 70% of patients [5–7]. Functional sequelae, persistent cognitive deficits and mental health problems can negatively affect health-related quality of life (HR-QoL) [8–10].

The assessment of HR-QoL has become a key issue in patient-reported outcome research. The SF-36 is a well-established tool for measuring multidimensional HR-QoL, representing patients’ perspectives of their health status, and providing eight health domains that can be divided into a physical component summary (PCS) and a mental component summary (MCS) [11]. HR-QoL is often reduced in the long term after SAH compared to the reference population [12–19]. Factors associated with reduced HR-QoL include worse clinical condition on admission, female sex, older age, physical disability, cognitive complaints and others [10]. Despite previous studies, it is still unclear how survivors across all levels of severity perceive their QoL and which concomitant factors influence QoL.

The current study aims to investigate 1) the prevalence of reduced HR-QoL as assessed by the SF-36 one year after SAH, 2) risk factors for reduced HR-QoL, and 3) co-occurring multidimensional outcome parameters associated with reduced HR-QoL.

## Methods

### Study design, setting, and participants

This observational, prospective cohort study, enrolled patients with a spontaneous SAH who were admitted to the neurological intensive care unit (ICU) of the Medical University of Innsbruck between April 2010 and October 2022. The study period was one year for each patient. Of 498 patients, 183 patients fulfilled the following inclusion criteria: (1) diagnosis of a spontaneous aneurysmal and non-aneurysmal SAH confirmed by computed tomography (CT) scan or lumbar puncture, (2) age greater or equal to 18 years, (3) ICU stay for more than 24 h, (4) fluent in the German language, and (5) multidimensional outcome evaluation including HR-QoL at the 1-year follow-up. The conduct of the study was approved by the hospital’s institutional review board (Medical University of Innsbruck, AM4091-292/4.6). Written informed consent was obtained from all patients according to local regulations, in accordance with the Declaration of Helsinki. Results are reported based on the Strengthening the Reporting of Observational Studies in Epidemiology statement.

### Clinical management

All patients were treated according to evidence-based international guidelines, except for nimodipine, which was administered intravenously in poor-grade patients [20–22]. All patients received prophylactic nimodipine. Ruptured aneurysms were treated by endovascular coiling or neurosurgical clipping based on interdisciplinary case-by-case discussion. Repeated transcranial color-coded duplex sonography (LOGIQ S8; GE Healthcare, Chicago, IL) was performed to detect sonographic large-vessel vasospasm, defined as an increase in mean velocities > 120 cm/s in the middle or anterior cerebral artery or a daily change in mean velocities > 50 cm/s. In cases of severe vasospasm, intra-arterial nimodipine was administered after confirmation by catheter cerebral angiogram. Delayed cerebral ischemia (DCI) was defined as neurological deterioration (new focal neurological deficits and/or a ≥ 2-point decline on the Glasgow Coma Scale) and/or new ischemic lesions on follow-up imaging (CT or magnetic resonance imaging) not attributable to other causes [23].

Patient demographics, disease-specific characteristics, treatment interventions, and hospital complications were prospectively recorded in our institutional SAH database and confirmed at weekly meetings of the study team and attending neurointensivists.

### Outcome instruments

Functional outcome was assessed at discharge, as well as 3 months and 12 months after SAH using the modified Rankin Scale (mRS). The mRS was used as a categorical (mRS ≤ 2 vs mRS > 2) or ordinal level variable and reported accordingly.

One year after SAH, patients underwent a structured interview, clinical examination, neuropsychological testing and completed self-report questionnaires. All of the following outcome instruments were completed one year after SAH:

HR-QoL as assessed by the SF-36 was the primary outcome measure of this study. The SF-36 is a self-report questionnaire that assesses the subjective health condition [11]. It provides scores for eight health domains: Physical functioning, physical role functioning, bodily pain, general health perceptions, vitality, social role functioning, emotional role functioning, and mental health. Each subscale ranges from 0 to 100 points, where higher scores imply better health conditions. The first four subscales are summarized into the physical component score (PCS). The last four subscales are summarized into a mental component summary (MCS). MCS and PCS were rescaled in relation to a 1994 norm-based German population, in which scores were standardised to 50 ± 10 (mean ± SD) [24]. By definition, a normal population has mean scores of 50 with standard deviations of 10. In the current study, norm-based T-scores below 40 were considered to be below the normative range. The Hospital Anxiety and Depression Scale (HADS; self-report questionnaire) was used to detect subjective symptoms of anxiety and depression in SAH survivors [25]. It consists of subscale for anxiety (HADS-A) and for depression (HADS-D), each of which includes seven items scored from 0 to 3. Scores on each subscale range from 0 to 21. Lower scores are associated with less severe anxiety and depression symptoms. Scores > 7 are suggestive of mild to clinically meaningful anxiety or depression disorder and were used for multivariable analysis. Scores > 10 indicate severe anxiety or depression disorder.

Subjectively perceived attention deficits in everyday situations were assessed with the FEDA (Fragebogen Erlebter Defizite der Aufmerksamkeit; self-report questionnaire) [26], which consists of three subscales (27 items): distractibility and retardation in mental tasks (FEDA-1; e.g., watching movies), tiredness and retardation in practical activities (FEDA-2; e.g., doing laundry), and possible reduction in drive (FEDA-3; e.g., interest in hobbies). The FEDA is standardized and scores ≤ 40/65 on the first, ≤ 27/40 on the second, and ≤ 17/30 on the third subscale are below the 10th percentile of the norm and were considered abnormal for the current study [27].

Neuropsychological testing was performed by experienced neuropsychologists, blinded to clinical outcome, one year after SAH using a standardised test battery as described previously. [5]. Executive function was assessed with six different tests: (1) the Frontal Assessment Battery (FAB) [28, 29]; (2) the semantic verbal fluency test (animals per minute)—Regensburger Verbal Fluency Test (Regensburger Wortflüssigkeitstest; RWT) to assess executive language functions [30]; the Trail Making Test (TMT) A (3) and B (4) to assess processing speed and mental flexibility [31]; (5) the digit span backwards subtest of the Wechsler Memory Scale revised assessing verbal working memory [32]; and (6) a clock drawing task (CLOX) to assess figure planning [33]. Executive function impairment was assumed if the patient scored below the 5th percentile (FAB, age-adjusted), the 10th percentile (according to age and/or education stratified norms) or below absolute values (CLOX < 11 points) on 2 out of 6 tests.

Two measures were used to assess memory function: (1) the delayed recall subtest of the Verbal Learning and Memory Test (Verbaler Lern- und Merkfähigkeitstest; VLMT) [34]; and (2) the delayed recall subtest of the Rey–Osterrieth complex figure test (ROCF) [35]. Impairment of memory function was assumed if the patient scored below the 10th percentile (according to age stratified norms) on 1 out of 2 tests.

Attention deficits were assumed if the patient scored below the 10th percentile (according to age norms) on the digit span forwards subtest of the Wechsler Memory Scale revised (WMS-R) [32].

Deficits in visuoconstruction were assessed using the copy subtest of the ROCF [35] and considered to be present if the patient scored below the 10th percentile.

### Statistical analysis

Categorical variables are presented as counts and percentages, and continuous variables are presented as medians and interquartile ranges or means and standard deviations. Based on the distribution of variables, parametric and non-parametric procedures including t-test, Mann–Whitney U test and Kruskal–Wallis test were used. Categorical variables were analyzed using chi-squared and Fisher’s exact test. The Wilcoxon rank test was used to assess differences in functional outcome over the one-year period. Two approaches were used to identify risk factors for reduced HR-QoL: 1) clinically relevant risk factors assessed during the acute phase of the disease were tested, 2) co-occurring multidimensional outcome factors assessed at one year were tested together with acute phase factors. Therefore, clinically meaningful and significantly associated factors (p < 0.1) in the univariate analysis were entered into a multivariable logistic regression model together with predefined covariates (Hunt&Hess, age). Backward elimination was used to remove nonsignificant factors until the best model with the lowest QIC (Quasilikelihood under the Independence model Criterion) values was obtained and the predictors were significant (p < 0.05). Overall reduced HR-QoL, PCS < 40 and MCS < 40 served as binary dependent variables. A p-value less than 0.05 was considered statistically significant. Statistical analysis was performed using IBM SPSS Statistics version 24 64-bit edition.

### Results

Of 498 patients admitted to our unit, 89 died in the ICU and a further 22 died during the first year, leaving 387 patients available for potential 1-year follow-up. Functional outcome (mRS) was available for 354 survivors and was comparable to 219 survivors who had detailed follow-up including neuropsychological testing one year after SAH (p = 0.117). Of these 219 patients, 183 completed the HR-QoL survey. The median age was 53 (IQR 46–61) years and 103 (56%) were female. Although there was a shift towards patients with good grades (median Hunt&Hess 2, IQR 2–3; Hunt&Hess 1–3: 84%), patients of all grades were included (Table 1). Included patients were generally younger, less severely affected, had fewer complications and better outcomes compared to 36 patients with detailed one-year outcomes who did not complete the SF-36 (Table 1).

**Table 1:** Subject demographics, baseline characteristics, hospital complications, and outcomes of included and excluded patients

			Included patients N = 183	Excluded patients N = 36	P-value^a^
**Baseline characteristics**					
Age, years			53 (46–61)	58 (52–68)	**0.007**
Sex, female			103 (56)	26 (72)	0.095
**Medical history**					
Arterial hypertension			66 (36)	17 (47)	0.259
Smoking history			77 (42)	14 (39)	0.854
Depression, medically treated			19 (10)	4 (11)	1.00
Anxiety, medically treated			6 (3)	2 (6)	0.622
**Admission variables**					
Admission Hunt & Hess score	1		75 (41)	7 (19)	**0.021**
	2		44 (24)	11 (31)	
	3		34 (19)	10 (28)	
	4		12 (7)	1 (3)	
	5		18 (10)	7 (19)	
Loss of consciousness at onset			44 (24)	17 (47)	**0.008**
Modified Fisher Score on admission			3 (2–4)	4 (3–4)	**0.015**
Intraparenchymal bleeding			22 (12)	5 (14)	0.783
SEBES			1 (0–2)	1 (0–2)	0.273
Intraventricular hemorrhage			84 (46)	25 (69)	**0.011**
Aneurysm			127 (69)	28 (78)	0.423
Perimesencephal SAH			28 (15)	5 (14)	1.000
**Aneurysm treatment**					
Coiling			87 (48)	21 (58)	0.651
Clipping			40 (22)	7 (19)	
No intervention (withhold therapy)			0 (0)	0 (0)	-
Withdrawn therapy during ICU stay			0 (0)	0 (0)	
**Hospital complications**					
Hydrocephalus requiring external ventricular drain			63 (34)	21 (58)	**0.009**
Mechanical ventilation during ICU stay			123 (67)	29 (81)	0.165
Ventilated days			1 (0–12)	10 (1–25)	**0.009**
Large-vessel vasospasm			82 (45)	19 (53)	0.465
Delayed cerebral ischemia			21 (12)	8 (22)	0.104
Sepsis/Bacteremia			13 (7)	8 (22)	**0.010**
Pneumonia			55 (30)	18 (50)	**0.032**
Ventriculitis			18 (10)	7 (19)	0.146
Urinary tract infection			43 (24)	12 (33)	0.214
**Outcomes**					
ICU length of stay, days			17 (9–25)	22 (17–38)	**0.005**
Discharge modified Rankin scale			2 (1–3)	4 (1–5)	** < 0.001**
3-month modified Rankin Scale			1 (0–2)	2 (1–4)	** < 0.001**
12-month modified Rankin Scale			1 (0–1)	2 (0–3)	** < 0.001**

Data are given in n (%) or median (IQR)

SEBES—Subarachnoid Hemorrhage Early Brain Edema; ICU – intensive care unit

^a^Differences across the included and excluded patients were calculated with the Mann Whitney U or the Fisher's exact test, as appropriate

Bold numbers signify statistical differences p<0.05

### HR-QoL 12 months after SAH

**Fig. 1:**
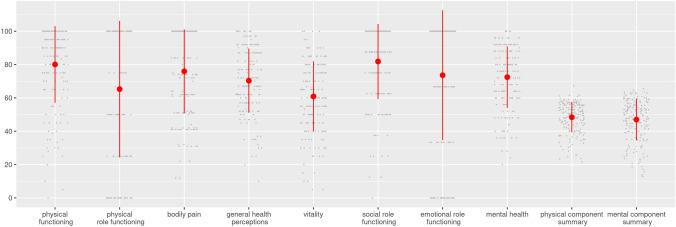
Mean (± SD) scores for physical component and mental component summary and subscales of the SF-36 survey

HR-QoL was reduced in 66/183 (36%), while 35 (19%) patients reported restrictions in the physical component summary, 48 (26%) in the mental component summary, and 17 (9%) in both components. Mean scores for both components were below population norms (physical component: 48.38 ± 9.04, p = 0.033; mental component: 46.99 ± 12.31, p < 0.001). Among the subscales, the highest mean difference from population norms was found for physical role (-17.70, p < 0.001), followed by emotional role (-16.38, p < 0.001), social functioning (-6.93, p < 0.001), and physical functioning (-5.50, p = 0.001). Bodily pain, general health, vitality, and mental health were within population norms (Fig. 1, Supplemental Table 1).

### Disease severity, ICU stay and HR-QoL after 12 months

Patients with a higher disease severity on admission (higher Hunt&Hess score) were more likely to have reduced physical QoL one year after SAH (PCS < 40, p = 0.040; Supplemental Table 2) but not MCS < 40 (p = 0.572). This association did not persist in multivariable analysis. Supplemental Table 2 shows univariate associations between patient characteristics, hospital complications, short-term outcomes and one-year HR-QoL.

Factors obtained at ICU stay that were associated with reduced overall HR-QoL in multivariable analysis included female sex [adjOR (95%-CI) 2.08 (1.06–4.11), p = 0.034] and a higher discharge mRS [per point, adjOR (95%CI) 1.51 (1.16–1.98), p = 0.029]. A higher mRS at discharge [per point, adjOR (95%CI) 1.86 (1.33–2.60), p < 0.001] was associated with reduced physical QoL (PCS < 40) in multivariable analysis. The only factor associated with reduced mental QoL (MCS < 40) in multivariable analysis was female sex [adjOR (95%CI) 2.22 (1.06–4.62), p = 0.034]. All models were adjusted for age and Hunt&Hess.

### Functional outcome and HR-QoL after 12 months

Median functional outcomes as assessed by the mRS improved from 2 (IQR 1–3) at discharge to 1 (IQR 0–2) at 3 months (p < 0.001) and further improved to 1 (IQR 0–1) at 1 year after SAH (p < 0.001). At discharge, 57% achieved a good functional outcome (mRS ≤ 2), 87% at 3 months and 96% at one year.

**Fig. 2:**
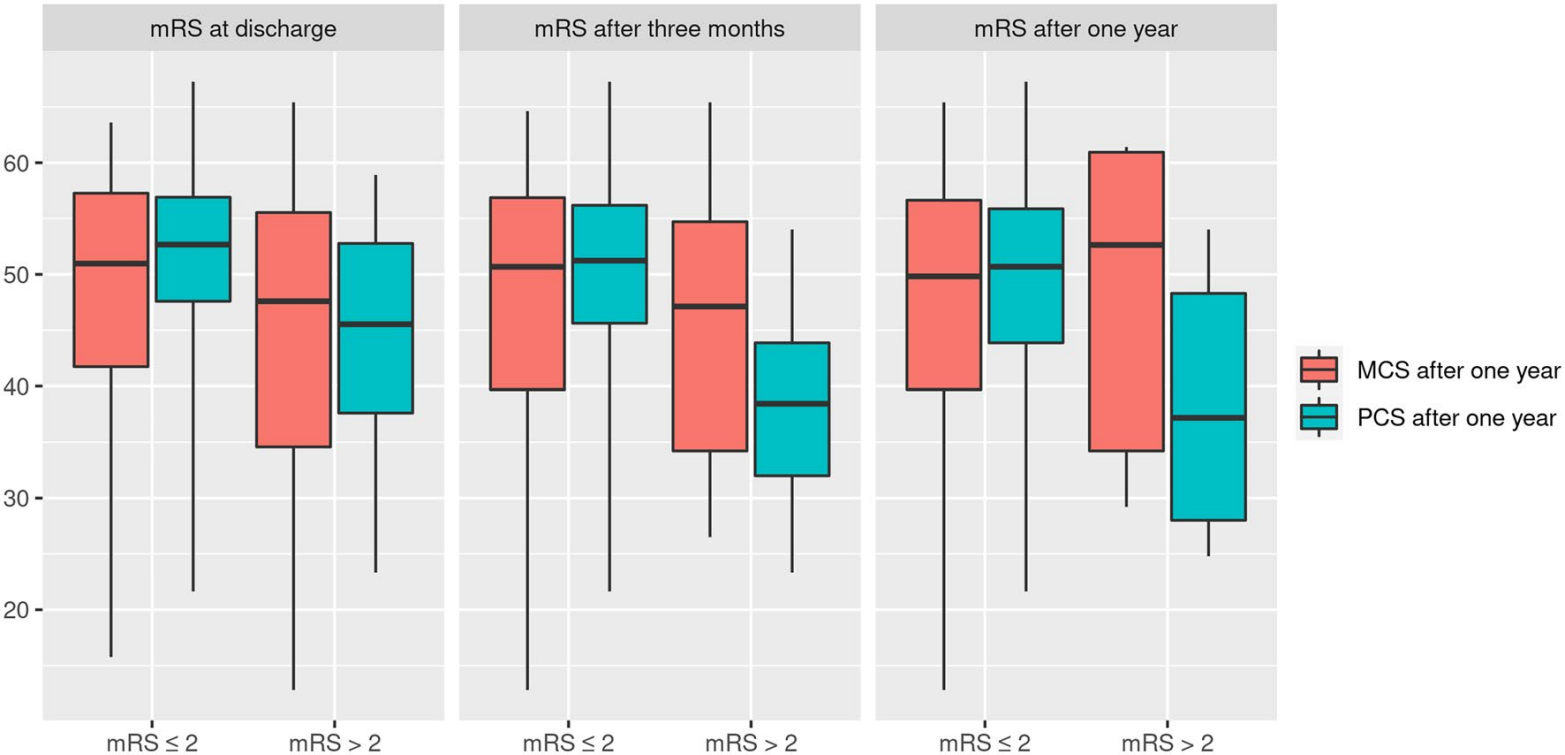
Box plots of physical component (PCS) and mental component summary (MCS) of the SF-36 at 1 year stratified by functional outcome as assessed by the modified Rankin Scale (mRS) at discharge, 3 months and 1 year. The central line shows the 50th percentile, the upper and lower lines the 75th and 25th percentile and the lines end at the 90th and 10th percentiles

Patients with poor functional outcome at discharge, 3 months and 12 months had lower PCS (p < 0.001, p < 0.001, p = 0.007, resp.) but not MCS scores at one year (p = 0.195, p = 0.520 and p = 0.668, resp., Fig. 2**).** A discordance between the PCS and MCS of almost 8 and 15 points was found for patients with poor 3 and 12-month functional outcomes, respectively.

### Cognitive outcome, mental health and HR-QoL after 12 months

Cognitive deficits at the 1-year follow-up, including executive deficits (p = 0.002) and memory deficits (p = 0.003), were more common in patients with reduced HR-QoL (Table 2).

At the one-year follow-up, 31/181 (17%) patients reported symptoms of depression and 61/181 (34%) reported symptoms of anxiety. Severe depression and anxiety (HADS > 10) were evident in 6% and 13% of patients, respectively. Self-reported symptoms of depression and anxiety were more common in patients with reduced HR-QoL including MCS and PCS < 40 (p = 0.001; Table 2).

Increased distractibility in everyday situations (FEDA-1 subtest) was reported by 20% of patients, increased tiredness (FEDA-2) by 39%, and a reduction of drive (FEDA-3) by 13%. All three symptoms were more common in patients with reduced HR-QoL including MCS and PCS < 40 (p = 0.001; Table 2).

**Table 2:** Univariate associations between one-year multidimensional outcomes and one-year health-related quality of life in 183 SAH patients

	MCS < 40	MCS ≥ 40	p-value	PCS < 40	PCS ≥ 40	p-value	MCS or PCS < 40	MCS or PCS ≥ 40	p-value
**Mental health impairments**									
HADS-A > 7	38 (79)	23 (17)	** < 0.001**	18 (51)	43 (30)	**0.017**	43 (65)	18 (16)	** < 0.001**
HADS-D > 7	24 (50)	7 (5)	** < 0.001**	12 (34)	19 (13)	**0.005**	27 (41)	4 (4)	** < 0.001**
HADS-A > 10	17 (35)	7 (5)	** < 0.001**	6 (17)	18 (12)	0.418	19 (30)	5 (4)	** < 0.001**
HADS-D > 10	10 (21)	1 (0.8)	** < 0.001**	5 (14)	6 (4)	**0.039**	11 (17)	0 (0)	** < 0.001**
HADS-A	9 (8–12)	4 (2–6)	** < 0.001**	8 (4–10)	5 (2–8)	**0.019**	8 (6–11)	4 (2–6)	** < 0.001**
HADS-D	8 (6–9)	2 (1–4)	** < 0.001**	6 (2–10)	3 (1–6)	** < 0.001**	6 (4–9)	2 (0–4)	** < 0.001**
FEDA-1 < 10th percentile	25 (52)	12 (9)	** < 0.001**	15 (43)	22 (15)	**0.001**	28 (42)	9 (8)	** < 0.001**
FEDA-2 < 10th percentile	40 (83)	32 (24)	** < 0.001**	33 (94)	39 (26)	** < 0.001**	56 (85)	16 (14)	** < 0.001**
FEDA-3 < 10th percentile	20 (43)	3 (2)	** < 0.001**	13 (37)	10 (7)	** < 0.001**	22 (34)	1 (1)	** < 0.001**
**Cognitive deficits**									
Any domain	36 (78)	69 (58)	**0.019**	26 (84)	79 (59)	**0.012**	49 (79)	56 (54)	**0.002**
Executive deficits	17 (36)	22 (18)	**0.024**	14 (44)	25 (18)	**0.004**	23 (37)	16 (15)	**0.002**
Visuoconstructive deficits	15 (32)	34 (25)	0.446	10 (29)	39 (27)	0.831	21 (32)	28 (24)	0.295
Memory deficits	29 (63)	51 (39)	**0.006**	19 (58)	61 (43)	0.127	39 (61)	41 (37)	**0.003**
Attention deficits	6 (13)	18 (13)	1.000	6 (18)	18 (12)	0.404	7 (11)	17 (15)	0.648
**Functional outcomes**									
mRS at 12 months	1 (1–2)	0 (0–1)	** < 0.001**	1 (1–2)	0 (0–1)	** < 0.001**	1 (1–2)	0 (0–1)	** < 0.001**

Mental component summary, *PCS *Physical component summary, *HADS* Hospital anxiety and Depression scale, *mRS *Modified Rankin scale score, *FEDA *Fragebogen Erlebter Defizite der Aufmerksamkeit, *FEDA-1 *Distractibility, *FEDA-2 *Tiredness, fatigue, *FEDA-3 *Reduction in drive

Data are given in n (%) or median (IQR). Univariate analysis was done with the Fisher’s exact test or Mann–Whitney U test, as appropriate

Bold numbers signify statistical differences p<0.05

### Multivariable analysis for HR-QoL

**Fig. 3:**
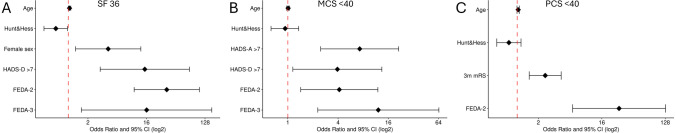
Factors associated with reduced **A** SF-36 < 40, **B** mental component summary (MCS) < 40, and **C** physical component summary (PCS) < 40 with calculated adjusted odds ratios based on the logistic regression with the 95% confidence intervals are shown. HADS A > 7 is indicative of the presence of anxiety, HADS D > 7 of depression; FEDA-2 < 10th percentile is indicative of tiredness and fatigue, FEDA-3 < 10th percentile of a reduction in drive. FEDA—Fragebogen Erlebter Defizite der Aufmerksamkeit

In multivariable analysis, including variables from the acute phase of the disease, 3-month and 12-month multidimensional outcome variables, a lower Hunt&Hess score at admission (p = 0.036), female sex (p = 0.017), self-reported depression (HADS-D > 7, p = 0.001), increased tiredness (FEDA-2, p < 0.001), and a reduction of drive (FEDA-3, p = 0.019) one year after SAH were associated with overall reduced HR-QoL and explained 68.9% of the variance (Table 3**, **Fig. 3). Self-reported anxiety and depression (HADS-A > 7, p < 0.001; HADS-D > 7, p = 0.029), increased tiredness (FEDA-2, p = 0.009), and a reduction of drive (FEDA-3 p = 0.003) at one year were associated with reduced MCS. These factors explained 61.7% of the variance in reduced MCS. Worse three-month functional outcome (higher mRS, p = 0.001) and increased tiredness at one year (FEDA-2, p < 0.001) were associated with reduced PCS, explaining 53.9% of the variance (Fig. 3). All models were adjusted for age and Hunt&Hess score on admission.

**Table 3:** Multivariable associations between patient characteristics, in-hospital complications, one-year multidimensional outcomes and one-year health-related quality of life in 183 SAH patients

	MCS < 40			PCS < 40			MCS or PCS < 40		
	adjOR	95% CI	p-value	adjOR	95% CI	p-value	adjOR	95% CI	p-value
Age	1.01	0.97–1.05	0.641	1.03	0.99–1.07	0.200	1.04	0.996–1.09	0.079
Hunt&Hess at admission	0.93	0.63–1.35	0.687	0.76	0.51–1.13	0.169	0.64	0.42–0.97	**0.036**
Sex, female							4.11	1.29–13.10	**0.017**
3-month mRS				2.50	1.48–4.22	**0.001**			
HADS-A > 7 at one year	7.27	2.48–21.29	** < 0.001**						
HADS-D > 7 at one year	3.93	1.15–13.39	**0.029**				15.09	3.09–73.57	**0.001**
FEDA-2 < 10th percentile at one year	4.14	1.43–12.00	**0.009**	28.17	6.13–129.40	** < 0.001**	32.89	10.33–104.77	** < 0.001**
FEDA-3 < 10th percentile at one year	12.14	2.29–64.53	**0.003**				16.10	1.59–162.69	**0.019**

*MCS *Mental component summary, *PCS *Physical component summary, *HADS *Hospital Anxiety and depression scale, *mRS *Modified Rankin scale score, *FEDA *Fragebogen Erlebter defizite der aufmerksamkeit, *FEDA-2 *Tiredness, fatigue, *FEDA-3 *Reduction in drive

With use of multivariable logistic regression analysis, factors associated with impaired one-year health-related quality of life were assessed. All models were adjusted for age and Hunt&Hess score on admission

Bold numbers signify statistical differences p<0.05

## Discussion

In this prospective observational study, we describe one-year HR-QoL assessed by the SF-36 of a large cohort of SAH patients. Overall HR-QoL was reduced in 36%, with 19% having a reduction in physical HR-QoL, and 26% in mental HR-QoL. Among subscales, physical and emotional role were the most abnormal compared to population norms. Mental health issues, fatigue and a reduction of drive mainly explained reduced mental HR-QoL, while worse functional outcome and fatigue was associated with reduced physical HR-QoL.

Reduced HR-QoL affected every third of our patients one year after SAH, which is in the lower range compared to a recent European study with SAH patients of similar severity and functional outcomes [36]. In another study, HR-QoL was reduced in 43% 4 months after SAH and the dimension most affected was physical role [37], which is consistent with our findings. Many studies show that QoL is reduced compared to population norms [12–19], but do not use cut-offs, making comparisons of prevalence difficult. Similarly, patients undergoing elective intracranial surgery [38], or those following treatment for unruptured intracranial aneurysms have a reduced HR-QoL in the long term [39].

Patient-reported QoL is a broad and multifaceted concept that encompasses both physical and mental aspects. The PCS of the SF-36 emphasizes aspects of functional status, while the MCS encompasses well-being, including mental health. Our study shows that impairments after SAH are multifactorial and affects several aspects of QoL, including physical role, emotional role, social functioning, and physical functioning. Similar to other studies we found that the physical and emotional roles are often the most affected dimensions of QoL after SAH [12, 14, 15, 40].

There are two points that need special attention. First, the patients included are mainly those who have recovered to a good functional outcome after one year. Despite the absence of severe functional sequelae, the prevalence of reduced HR-QoL was relatively high, showing a divergent pattern of good physical functioning but problems in the emotional and social domains. This highlights the importance of screening for QoL measures that may interact with daily living, social life, and ability to return to work, also in physically independent patients. On the other hand, a subanalysis of patients with poor functional outcomes revealed a discordance between the PCS and the MCS, confirming that individuals’ perceptions of aspects of well-being and mental health are often discordant with their objective functioning [41].

The second objective of the study was to identify factors associated with reduced QoL. Women were more likely to have reduced long-term HR-QoL and specifically reduced MCS, consistent with previous work [14, 37, 42]. One explanation may be that even healthy women report lower HR-QoL measures than men [43]. Furthermore, women are known to have significantly lower odds of good clinical outcomes after SAH despite similar clinical severity, which may influence HR-QoL [44]. Also, female patients are more likely to report anxiety, depression and fatigue, which are strongly associated with reduced health-related QoL [40]. Research shows that female patients are at higher risk of cognitive deficits after SAH, which is a significant factor affecting QoL, particularly in women. [45]. In our data, we could only find higher odds for anxiety in women, independent of disease severity, but not for functional outcome, depression or fatigue.

We could not replicate an association between poor clinical admission grade and reduced HR-QoL in multivariable analysis [10, 14, 46], but actually found an independent association between better clinical admission grade and reduced overall HR-QoL, which was also found in one previous study [47]. One explanation might be that a higher H&H score was associated with poor functional outcome at all time-points (p < 0.001, data not shown) and poor functional outcome in turn was associated with lower PCS but not MCS, consistent with a meta-analysis [48]. This highlights the concept of disability paradox: patients with severe disease and poor functional outcome still have good QoL, which is known from previous work [41]. Conversely, patients with favourable initial clinical grades, despite good functional recovery, may still have subtle deficits that significantly affect their QoL, and may even affect their self-perceived QoL more than a patient with a higher grade SAH would have felt.

Fatigue was a determinant of reduced PCS and MCS. Fatigue after SAH can have two major aspects, namely mental and physical fatigue [49], which explains its impact on mental and physical HR-QoL. It is an important symptom after SAH [50] and has already been shown to have a significant impact on HR-QoL [40, 49, 51], emphasizing the importance of managing the symptom of fatigue. Currently, there are no pharmacological therapies to improve fatigue after stroke [52], but some non-pharmacological strategies may improve fatigue symptoms [53]. Multidisciplinary health management, including medication management, fatigue education, community activities, and psychological care, is a promising approach [53].

Although fatigue is a relevant symptom in the clinical assessment of depression, we tested both because they are distinct poststroke symptoms and the HADS does not include questions about fatigue. Together with self-reported anxiety and a decreased drive, these four factors explained 61.7% of the variance in reduced MCS, which is consistent with previous findings [40]. This suggests that problems in the emotional domains have a major impact on SAH survivors. In this regard, it is important to pay special attention to mental health symptoms even in physically independent patients, as depression and anxiety in particular can be treated.

Some limitations of this study deserve mention. Firstly, we did not assess HR-QoL before or shortly after SAH and cannot conclude on the temporal evolution and natural history of QoL. Secondly, we used a German reference population from 1994, which may be outdated. Self-perceived HR-QoL increased slightly over time, so we probably underestimated the true prevalence of reduced HR-QoL [43]. Thirdly, there may have been a selection bias towards patients with good functional recovery. However, functional outcomes were generally good in survivors, even in patients who did not undergo detailed one-year follow-up or QoL assessment (median mRS for 354/387 survivors: 1, IQR 0–2, good functional outcome 84%). 22% of all SAH patients died within one year and QoL could not be assessed. Fourthly, backward elimination in multivariable modelling may have led to overfitting with reduced generalisability to other data sets [54].

## Conclusion

Our study suggests that self-perceived HR-QoL is reduced in the long term after SAH despite good functional recovery. The high impact of mental health deficits calls for a thorough screening of mental health in SAH patients in order to provide a more tailored, multidisciplinary rehabilitation program aimed at improving patients' QoL.

## Supplementary Information

Below is the link to the electronic supplementary material.

Supplementary file1 (DOCX 22 KB)

## Data Availability

The data that support the findings of this study are available from the corresponding author, upon reasonable request.
