# Supplementary material for: Health-related quality of life after spontaneous subarachnoid hemorrhage – a prospective cohort study
Source: Qual Life Res. 2025 Mar 27;34(7):1955–65. doi: 10.1007/s11136-025-03955-6 (PMC12182521; doi:10.1007/s11136-025-03955-6)
Supplement: Supplementary file 1 — Supplementary file1 (DOCX 22 KB) [file 11136_2025_3955_MOESM1_ESM.docx]

| **Supplemental Table 1. Scores of the SF-36 survey compared against normal values.** | | | | |
| --- | --- | --- | --- | --- |
|  | Mean (±SD) | Mean difference, 95%-CI | p-value | Normal values, Germany 1994 |
| Physical Component Summary (PCS) | 48.38±9.04 | -1.62, -3.11 to -0.13 | **0.033** | 50±10; ≥40 |
| Mental Component Summary (MCS) | 46.99±12.31 | -3.01, -4.53 to -1.49 | **<0.001** | 50±10; ≥40 |
| Physical Functioning (PF) | 80.06±23.00 | -5.50, -8.84 to -2.16 | **0.001** | 85.56±22.32 |
| Role Physical (RP) | 65.26±40.91 | -17.70, -22.65 to-12.75 | **<0.001** | 82.96±32.59 |
| Bodily Pain (BP) | 75.94±25.15 | -3.14, -7.21 to 0.93 | 0.130 | 79.08±27.36 |
| General Health (GH) | 70.31±19.22 | 2.39, -0.63 to 5.41 | 0.128 | 67.92±20.27 |
| Vitality (VT) | 60.87±21.03 | -2.34, -5.12 to 0.44 | 0.100 | 63.21±18.46 |
| Social Functioning (SF) | 81.83±22.49 | -6.93, -9.72 to -4.14 | **<0.001** | 88.76±18.39 |
| Role Emotional (RE) | 73.59±38.92 | -16.38, -20.44 to -12.32 | **<0.001** | 89.97±26.25 |
| Mental Health (MH) | 72.44±18.43 | -1.32, -3.81 to 1.17 | 0.299 | 73.76±16.57 |

| **Supplemental Table 2: Univariate associations between patient characteristics, hospital complications, short-term outcomes and one-year health-related quality of life in 183 SAH patients.** | | | | | | | | | |
| --- | --- | --- | --- | --- | --- | --- | --- | --- | --- |
|  | **MCS <40** | **MCS ≥40** | **p-value** | **PCS <40** | **PCS ≥40** | **p-value** | **MCS or PCS <40** | **MCS or PCS ≥40** | **p-value** |
| **Baseline characteristics** | | | | | | | | | |
| Age, years | 53 (44-60) | 53 (46-61) | 0.656 | 55 (46-66) | 52 (46-60) | 0.087 | 54 (47-63) | 52 (46-60) | 0.259 |
| Sex, female | 34 (71) | 69 (51) | **0.019** | 24 (69) | 79 (53) | 0.130 | 46 (70) | 57 (49) | **0.008** |
| **Admission variables** | | | | | | | | | |
| Admission Hunt & Hess score | 2 (1-3) | 2 (1-3) | 0.572 | 3 (1-3) | 2 (1-3) | **0.040** | 2 (1-3) | 2 (1-3) | 0.161 |
| Loss of consciousness at ictus | 11 (23) | 33 (24) | 1.000 | 13 (37) | 31 (21) | 0.050 | 18 (27) | 26 (22) | 0.474 |
| Modified Fisher Score on admission | 3 (1-4) | 3 (2-4) | 0.144 | 3 (2-4) | 3 (2-4) | 0.074 | 3 (2-4) | 3 (2-4) | 0.801 |
| Intraparenchymal bleeding | 7 (15) | 15 (11) | 0.608 | 5 (14) | 17 (12) | 0.773 | 8 (12) | 14 (12) | 1.000 |
| SEBES score | 1 (0-2) | 1 (0-2) | 0.932 | 1 (0-2) | 1 (0-2) | 0.635 | 1 (0-2) | 0 (0-2) | 0.900 |
| Intraventricular hemorrhage | 20 (42) | 64 (47) | 0.506 | 22 (63) | 62 (42) | **0.037** | 32 (49) | 52 (44) | 0.645 |
| Aneurysm | 35 (73) | 92 (68) | 0.588 | 28 (80) | 99 (67) | 0.156 | 50 (76) | 77 (66) | 0.184 |
| Perimesencephal SAH | 8 (17) | 20 (15) | 0.816 | 4 (11) | 24 (16) | 0.607 | 9 (14) | 19 (16) | 0.676 |
| **Aneurysm treatment** | | | | | | | | | |
| **Aneurysm** |  |  |  |  |  |  |  |  |  |
| Coiling (vs Clipping) | 24 (69) | 63 (69) | 1.000 | 20 (74) | 67 (67) | 0.641 | 34 (69) | 53 (68) | 1.000 |
| **Hospital complications** | | | | | | | | | |
| Hydrocephalus requiring external ventricular drain | 14 (29) | 49 (36) | 0.480 | 16 (46) | 47 (32) | 0.165 | 24 (36) | 39 (33) | 0.747 |
| Ventilated days | 2 (0-14) | 1 (0-12) | 0.579 | 3 (0-16) | 1 (0-12) | 0.201 | 3 (0-14) | 1 (0-12) | 0.216 |
| Large-vessel vasospasm | 24 (50) | 58 (43) | 0.405 | 16 (46) | 66 (45) | 1.000 | 31 (47) | 51 (44) | 0.757 |
| Delayed cerebral ischemia | 8 (17) | 13 (10) | 0.196 | 6 (17) | 15 (10) | 0.246 | 11 (17) | 10 (9) | 0.145 |
| Pneumonia | 16 (33) | 39 (29) | 0.586 | 14 (40) | 41 (28) | 0.158 | 24 (36) | 31 (27) | 0.181 |
| Ventriculitis | 6 (13) | 12 (9) | 0.572 | 3 (9) | 15 (10) | 1.000 | 7 (11) | 11 (9) | 0.800 |
| Sepsis/Bacteremia | 3 (6) | 10 (7) | 1.000 | 2 (6) | 11 (7) | 1.000 | 4 (6) | 9 (8) | 0.773 |
| **Functional outcomes** | | | | | | | | | |
| ICU length of stay, days | 18 (9-28) | 16 (9-25) | 0.613 | 23 (13-38) | 15 (9-24) | **0.011** | 20 (10-29) | 15 (9-25) | 0.073 |
| Discharge modified Rankin scale | 3 (1-3) | 2 (1-3) | 0.144 | 3 (2-4) | 1 (1-3) | **<0.001** | 3 (1-4) | 1 (1-3) | **0.001** |
| 3-month modified Rankin Scale | 1 (1-2) | 1 (0-2) | **<0.001** | 2 (1-3) | 1 (0-1) | **<0.001** | 2 (1-3) | 1 (0-1) | **<0.001** |
| ICU – intensive care unit; MCS – mental component summary; PCS – physical component summary; SEBES - Subarachnoid Hemorrhage Early Brain Edema; mRS – modified Rankin Scale Score  Data are given in n (%) or median (IQR). Univariate analysis was done with the Fisher’s exact test or Mann-Whitney U test, as appropriate. | | | | | | | | | |
